# Supplementary material for: Orbital torque in magnetic bilayers
Source: Nat Commun. 2021 Nov 18;12:6710. doi: 10.1038/s41467-021-26650-9 (PMC8602295; doi:10.1038/s41467-021-26650-9)
Supplement: Supplementary file 1 — Supplementary Information [file 41467_2021_26650_MOESM1_ESM.pdf]

Supplementary Information for

Orbital torque in magnetic bilayers

Dongjoon Lee, Dongwook Go, Hyeon-Jong Park, Wonmin Jeong, Hye-Won Ko, Deokhyun Yun, Daegeun Jo, Soogil Lee, Gyungchoon Go, Jung Hyun Oh, Kab-Jin Kim, Byong-Guk Park, Byoung-Chul Min, Hyun Cheol Koo, Hyun-Woo Lee, OukJae Lee, and Kyung-Jin Lee

Section S1. First-principles calculation

Section S2. FM-thickness dependence of ST-FMR signals

Section S3. Linewidth (*ΔH*) modulation by a DC current

Section S4. In-plane 2nd harmonic Hall measurements

Section S5. Discussion on the SOC in 3d-FM

Section S6. Overall trends of the SOT variation

Fig. S1. Comparison between $\langle \delta S_x \rangle$ and $\langle \delta S_x \rangle^{\mathrm{OT,bulk}}$.

Fig. S2. Spin-orbit correlation $R_{FM} = \langle L \cdot S \rangle_{FM}$ for various FMs (Co, Fe, CoFe, and Ni).

Fig. S3. $V_{mix}(H)$ of Ni(t_{Ni})/Pt bilayers.

Fig. S4. $V_{mix}(H)$ of Ni(t_{Ni})/Ta bilayers.

Fig. S5. $V_{mix}(H)$ of Ni(7)/Cu(t_{Cu})/Ta layers.

Fig. S6. $V_{mix}(H)$ of Ni(t_{Ni})/Cu(1)/Ta layers.

Fig. S7. $V_{mix}(H)$ of HfO_x/Ni(t_{Ni})/Pt layers.

Fig. S8. $V_{mix}(H)$ of HfO_x/Ni(t_{Ni})/Ta layers.

Fig. S9. Linewidth (*ΔH*) modulation by a DC current.

Fig. S10. $R_H^{2\omega}$(φ) of FM/NM layers.

Fig. S11. R_H^{ω}(Hz) of FM/NM layers.

Fig. S12. Estimation of SOTs from 2nd harmonic Hall measurements.

Fig. S13. Evaluated Landé g-factors

Fig. S14. The trend of SOT variation among various FM/NM bilayers

Section S1. First-principles calculation

The self-consistent calculation of the electronic structure was carried out from the density functional theory code FLEUR[1], which implements the full-potential linearized augmented plane wave method[2]. We employed the Perdew-Burke-Ernzerhof exchange-correlation functional within the generalized gradient approximation[3]. For NMs, we assumed face-centered cubic (fcc) structure for Pt (a=7.52a_0), tetragonal (distorted A15) structure for β-Ta (a=10.09a_0, c=9.39a_0), where a_0 is the Bohr radius. The muffin-tin radii for NM atoms were chosen as 2.59a_0, 2.65a_0, and 2.29a_0 for fcc Pt and β-Ta, respectively. All FMs were assumed to have the fcc structure except for CoFe, which is assumed to have the simple cubic (sc) structure. Lattice constants of Fe, Co, Ni, and CoFe were set a=6.89a_0, a=6.67a_0, a=6.65a_0, and a=5.37a_0 respectively. The muffin-tin radii of the FM atoms were chosen as 2.37a_0, 2.30a_0, and 2.29a_0 for Fe, Co, and Ni, respectively. For CoFe, muffin-tin radii of Co and Fe are both set 2.26a_0. For all ferromagnets, the magnetization direction is set along the z-direction. The Monkhorst-Pack **k**-mesh of $16 \times 16 \times 16$ was sampled from the first Brillouin zone for all the elements.

Based on the Bloch states obtained from the converged charge density, maximally-localized Wannier functions (MLWFs) were obtained from the code package WANNIER90[4]. For all NMs and FMs, we constructed 18 MLWFs for each atom in the unit cell by initially projecting to *s*, *p*, and *d* states, starting from the Bloch states whose number is two times larger than the number of the MLWFs. The upper bound of the frozen window for the disentanglement was set to 6 eV above the Fermi energy. Then representations of the Hamiltonian, spin, and orbital operators were evaluated within the basis set of MLWFs, which are Fourier-transformed into interpolated **k**-mesh of $200 \times 200 \times 200$. The orbital angular momentum operator was evaluated within the muffin-tin sphere of each atom. The orbital Hall conductivity (σ_{OH}^{NM}) and

the spin Hall conductivity (σ_{SH}^{NM}) of NM=Pt, Ta were integrated over the interpolated **k**-mesh. The resulting values are given in Fig.2A.

The σ_{OH}^{NM} and σ_{SH}^{NM} were evaluated from the Kubo formula

$$\sigma_{\mathrm{OH(SH)}} = -e\hbar \sum_{n,m} \int \frac{d^3k}{(2\pi)^3} (f_{n\boldsymbol{k}} - f_{m\boldsymbol{k}}) \,\mathrm{Im}\left[\frac{\langle u_{n\boldsymbol{k}} | j_z^{X_y}(\boldsymbol{k}) | u_{m\boldsymbol{k}}\rangle \langle u_{m\boldsymbol{k}} | v_x(\boldsymbol{k}) | u_{n\boldsymbol{k}}\rangle}{(E_{n\boldsymbol{k}} - E_{m\boldsymbol{k}} + i\Gamma)^2}\right], \quad \text{(S1)}$$

where the level broadening Γ is set to 25 meV (room temperature scale), $e > 0$ is the elementary charge, $\hbar$ is the Planck constant divided by 2π, $f_{n\boldsymbol{k}}$ is the Fermi-Dirac distribution function for a periodic part of the Bloch state $|u_{n\boldsymbol{k}}\rangle$ with its energy eigenvalue $E_{n\boldsymbol{k}}$. For the velocity operator $\boldsymbol{v}(\boldsymbol{k}) = (1/\hbar)\nabla H(\boldsymbol{k})$, the z-component of the orbital (spin) current operator is defined as

$$j_z^{X_y}(\boldsymbol{k}) = \frac{1}{2}\left[v_z(\boldsymbol{k})X_y + X_y v_z(\boldsymbol{k})\right] \quad \text{(S2)}$$

for y-component of the orbital (spin) angular momentum $X_y = L_y(S_y)$.

When an orbital Hall current is injected into FM in addition to a spin Hall current, both the orbital and spin Hall currents can generate spin accumulation $\langle \delta\mathbf{S}\rangle$ in FM, where $\langle \delta\mathbf{S}\rangle$ denotes the integrated spin density over the volume of FM and is related to the total torque **T** in the FM,

$$\mathbf{T} = \frac{J}{\hbar}\hat{\mathbf{m}} \times \langle \delta\mathbf{S}\rangle . \quad \text{(S3)}$$

Here J is the exchange coupling constant in FM, and the unit vector $\hat{\mathbf{m}}$ denotes the direction of the magnetization in FM. When $\hat{\mathbf{m}}$ is pointing along the z-direction (out-of-plane direction of a bilayer) and an external electric field is applied along the x-direction, which is the case assumed here, the damping-like component of **T** is determined by the x component $\langle \delta S_x\rangle = \langle \delta\mathbf{S}\rangle \cdot \hat{\mathbf{x}}$ of $\langle \delta\mathbf{S}\rangle$.

Here, we aim to estimate the contributions to $\langle \delta S_x\rangle$ generated from the two types of injection separately; the orbital Hall current injection and the spin Hall current injection. Explicit first-

principles-based evaluation of the two individual contributions for real FM/NM bilayers is quite challenging computationally. For this purpose, we devise a scheme to estimate the two individual contributions separately from the bulk properties of NM and FM, of which first-principles calculation is much less demanding. This bulk-property-based evaluation scheme is tested for a model FM/NM bilayers and found to produce reasonable estimations as described below.

The contribution to $\langle \delta S_x \rangle$ from the orbital Hall current injection is estimated as $\langle \delta S_x \rangle^{\mathrm{OT,bulk}} = T_{\mathrm{L}} \sigma_{\mathrm{OH}}^{\mathrm{NM}} \chi_{\mathrm{LS}}^{\mathrm{FM}}$ where the superscript "OT, bulk" is introduced in $\langle \delta S_x \rangle^{\mathrm{OT,bulk}}$ to denote that this contribution is evaluated mostly from bulk properties of NM and FM: The orbital Hall conductivity σ_{OH}^{NM} is evaluated for bulk NM, and the orbital-spin susceptibility χ_{LS}^{FM} (precise definition given below) is evaluated for bulk FM. Thus, the only parameter in $\langle \delta S_x \rangle^{\mathrm{OT,bulk}}$ that depends on the existence of the FM/NM interface is the positive proportionality factor T_{L} that describes the degree of the orbital current transmission from NM to FM.

To be more specific, the orbital Hall conductivity σ_{OH}^{NM} is calculated for bulk NM by using the Kubo formula in Equation (S1). On the other hand, the orbital-spin susceptibility χ_{LS}^{FM} is calculated for bulk FM by using the following Kubo formula,

$$\chi_{\mathrm{LS}}^{\mathrm{FM}} = \frac{e}{\hbar} \sum_{n,m} \int \frac{d^3 k}{(2\pi)^3} \left(f_{n\mathbf{k}} - f_{m\mathbf{k}} \right) \mathrm{Re} \left[\frac{\langle u_{n\mathbf{k}} | S_x | u_{m\mathbf{k}} \rangle \langle u_{m\mathbf{k}} | L_x | u_{n\mathbf{k}} \rangle}{\left(E_{n\mathbf{k}} - E_{m\mathbf{k}} + i\Gamma \right)^2} \right], \tag{S4}$$

where the level broadening Γ is set to 25 meV (room temperature scale). The orbital-spin susceptibility χ_{LS}^{FM} defined by this formula quantifies how much spin accumulation is induced when an external perturbation $H' = \frac{L_x}{\hbar} e V_{\mathrm{L}}$

V_{L} may be regarded as the orbital-dependent voltage. Thus the combination $T_{\mathrm{L}}\sigma_{\mathrm{OH}}^{\mathrm{NM}}\chi_{\mathrm{LS}}^{\mathrm{FM}}$ in $\langle \delta S_x \rangle^{\mathrm{OT,bulk}}$ has the following physical meaning:

$$\begin{aligned} T_{\mathrm{L}}\sigma_{\mathrm{OH}}^{\mathrm{NM}}\chi_{\mathrm{LS}}^{\mathrm{FM}} \sim & \left(\sigma_{\mathrm{OH}}^{\mathrm{NM}} \sim \text{degree of orbital current generation in NM}\right) \\ & \times\left(T_{\mathrm{L}} \sim \text{degree of orbital current transmission into FM}\right) \\ & \times\left(\chi_{\mathrm{LS}}^{\mathrm{FM}} \sim \text{degree of conversion from orbital to spin in FM}\right) \\ \sim & \left(\text{degree of spin accumulation generation in FM by orbital Hall effect in NM}\right). \end{aligned}$$

Before we apply this estimation scheme to real bilayers, we test this estimation scheme for a model system in Ref. (*20*), where NM is modeled as a *sp* metal in a simple cubic lattice without SOC and FM is modeled as a *d* metal in a simple cubic lattice. The atomic ordering at the FM/NM interface is assumed to be perfect. For this model bilayer structure, the spin Hall current injection does not occur, and the angular momentum injection arises entirely from the orbital Hall current. Since the model system is less demanding in terms of the numerical calculation, we evaluate the orbital Hall current injection contribution to $\langle \delta S_x \rangle$ explicitly by taking into account the entire bilayer system (that is, without making the bulk approximation). The result is compared to its bulk approximation $\langle \delta S_x \rangle^{\mathrm{OT,bulk}} = T_{\mathrm{L}}\sigma_{\mathrm{OH}}^{\mathrm{NM}}\chi_{\mathrm{LS}}^{\mathrm{FM}}$. To be more specific, we compare the E_{F}-dependence of $\langle \delta S_x \rangle$ with the E_{F}-dependence of $\langle \delta S_x \rangle^{\mathrm{OT,bulk}} = T_{\mathrm{L}}\sigma_{\mathrm{OH}}^{\mathrm{NM}}\chi_{\mathrm{LS}}^{\mathrm{FM}}$. For this comparison, T_{L} is assumed to be independent of E_{F} and regarded as a fitting parameter. The result is shown in Fig. S1. The solid black line denotes $\langle \delta S_x \rangle^{\mathrm{OT,bulk}}$ and the red square symbols the explicitly calculated $\langle \delta S_x \rangle$ for the bilayer. For the calculation of $\langle \delta S_x \rangle$, the NM is assumed to have 8 atomic layers and the FM 2 atomic layers. We find that $\langle \delta S_x \rangle^{\mathrm{OT,bulk}}$ and $\langle \delta S_x \rangle$ agree well, which confirms that the orbital

torque calculated by extracting bulk parameters of the NM and FM provides a reasonable estimation.

To assess the total DLT for real material bilayers, the estimation of the spin Hall current contribution is also needed. We estimate this contribution as $\langle \delta S_x \rangle^{\mathrm{ST,bulk}} = T_{\mathrm{S}} \sigma_{\mathrm{SH}}^{\mathrm{NM}} \chi_{\mathrm{SS}}^{\mathrm{FM}}$ where the superscript "ST, bulk" is introduced in $\langle \delta S_x \rangle^{\mathrm{ST,bulk}}$ to denote that this estimation of the conventional spin-orbit torque is evaluated mostly from bulk properties of NM and FM: The spin Hall conductivity σ_{SH}^{NM} for bulk NM is calculated from the Kubo formula, Eq. (S1), and the spin-spin susceptibility χ_{SS}^{FM} for bulk FM, which quantifies what portion of the injected spin Hall current results in the spin accumulation in bulk FM, is calculated from the following Kubo formula,

$$\chi_{\mathrm{SS}}^{\mathrm{FM}} = \frac{e}{\hbar} \sum_{n,m} \int \frac{d^3k}{(2\pi)^3} \left(f_{n\mathbf{k}} - f_{m\mathbf{k}} \right) \mathrm{Re} \left[\frac{\langle u_{n\mathbf{k}} | S_x | u_{m\mathbf{k}} \rangle \langle u_{m\mathbf{k}} | S_x | u_{n\mathbf{k}} \rangle}{\left(E_{n\mathbf{k}} - E_{m\mathbf{k}} + i\Gamma \right)^2} \right], \tag{S5}$$

which is the linear response coefficient for the spin accumulation as a response to the perturbation $H' = \frac{S_x}{\hbar} e V_{\mathrm{S}}$ that enforces the spin accumulation through the spin-dependent voltage V_{S}. The only parameter in $\langle \delta S_x \rangle^{\mathrm{ST,bulk}}$ that depends on the existence of the FM/NM interface is the positive proportionality factor T_{S} that describes the degree of the spin current transmission from NM to FM. The combination $T_{\mathrm{S}} \sigma_{\mathrm{SH}}^{\mathrm{NM}} \chi_{\mathrm{SS}}^{\mathrm{FM}}$ in $\langle \delta S_x \rangle^{\mathrm{ST,bulk}}$ is the spin counterpart of the combination $T_{\mathrm{L}} \sigma_{\mathrm{OH}}^{\mathrm{NM}} \chi_{\mathrm{LS}}^{\mathrm{FM}}$ in $\langle \delta S_x \rangle^{\mathrm{OT,bulk}}$.

Hence the total spin accumulation $\langle \delta S_x \rangle$ for the DLT is estimated as the sum of the spin Hall and orbital Hall contributions:

$$\begin{aligned} \langle \delta S_x \rangle^{\mathrm{bulk}} &= \langle \delta S_x \rangle^{\mathrm{ST,bulk}} + \langle \delta S_x \rangle^{\mathrm{OT,bulk}} = T_{\mathrm{S}} \sigma_{\mathrm{SH}}^{\mathrm{NM}} \chi_{\mathrm{SS}}^{\mathrm{FM}} + T_{\mathrm{L}} \sigma_{\mathrm{OH}}^{\mathrm{NM}} \chi_{\mathrm{LS}}^{\mathrm{FM}} \\ &= T_{\mathrm{S}} \chi_{\mathrm{SS}}^{\mathrm{FM}} \left(\sigma_{\mathrm{SH}}^{\mathrm{NM}} + C_{\mathrm{FM}} \sigma_{\mathrm{OH}}^{\mathrm{NM}} \right) \end{aligned}. \tag{S6}$$

Here $C_{FM} = (T_L/T_S)(\chi_{LS}^{FM}/\chi_{SS}^{FM})$. The second factor $\chi_{LS}^{FM}/\chi_{SS}^{FM}$ can be evaluated from Eqs. (S4) and (S5), and the first factor T_L/T_S is assumed to be 0.3, which implies that the orbital injection is less efficient than the spin injection. This assumption is supported by a recent first-principles calculation of similar systems Fe/*β*-W and Ni/*β*-W (*39*), which deals with the bilayer systems explicitly without using the bulk approximation. The resulting value of C_{FM} is shown in Fig. 2B of the main text. Based on Eq. (S6), we define the effective spin Hall conductivity as $\sigma_{SH}^{NM} + C_{FM}\sigma_{OH}^{NM}$, of which value is presented in Figs. 2C and 2D for various Pt-based and Ta-based bilayers, respectively. Note that when σ_{SH}^{NM} and σ_{OH}^{NM} have opposite signs, which is the case for Ta, and when C_{FM} is sufficiently large, the sign of the effective spin Hall conductivity can differ from the sign of σ_{SH}^{NM}. Thus the abnormal sign of the effective spin Hall conductivity (also the abnormal sign of the DLT) is most likely to be achieved for the FM material with large C_{FM}, which turns out be Ni among conventional 3*d* FMs.

To understand the FM-material-dependent systematic variation of C_{FM} (Fig. 2B), it is illustrative to consider the spin-orbit correlation $R_{FM} = \langle \boldsymbol{L} \cdot \boldsymbol{S} \rangle_{FM}$ of FM. Among the four FMs (Co, Fe, CoFe, and Ni) examined, Ni has the strongest correlation near the Fermi energy (Fig. S2).

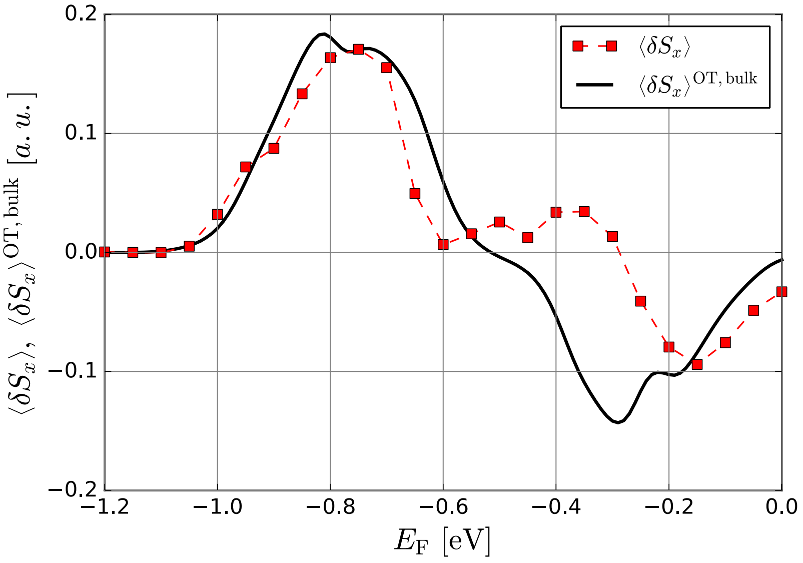

Fig. S1. Comparison between $\langle \delta S_x \rangle$ and $\langle \delta S_x \rangle^{\mathrm{OT,bulk}}$. Explicitly calculated $\langle \delta S_x \rangle$ (red square symbols) versus its bulk approximation by $\langle \delta S_x \rangle^{\mathrm{OT,bulk}}$ (black solid line) for the model FM/NM bilayer system in Ref. (*20*).

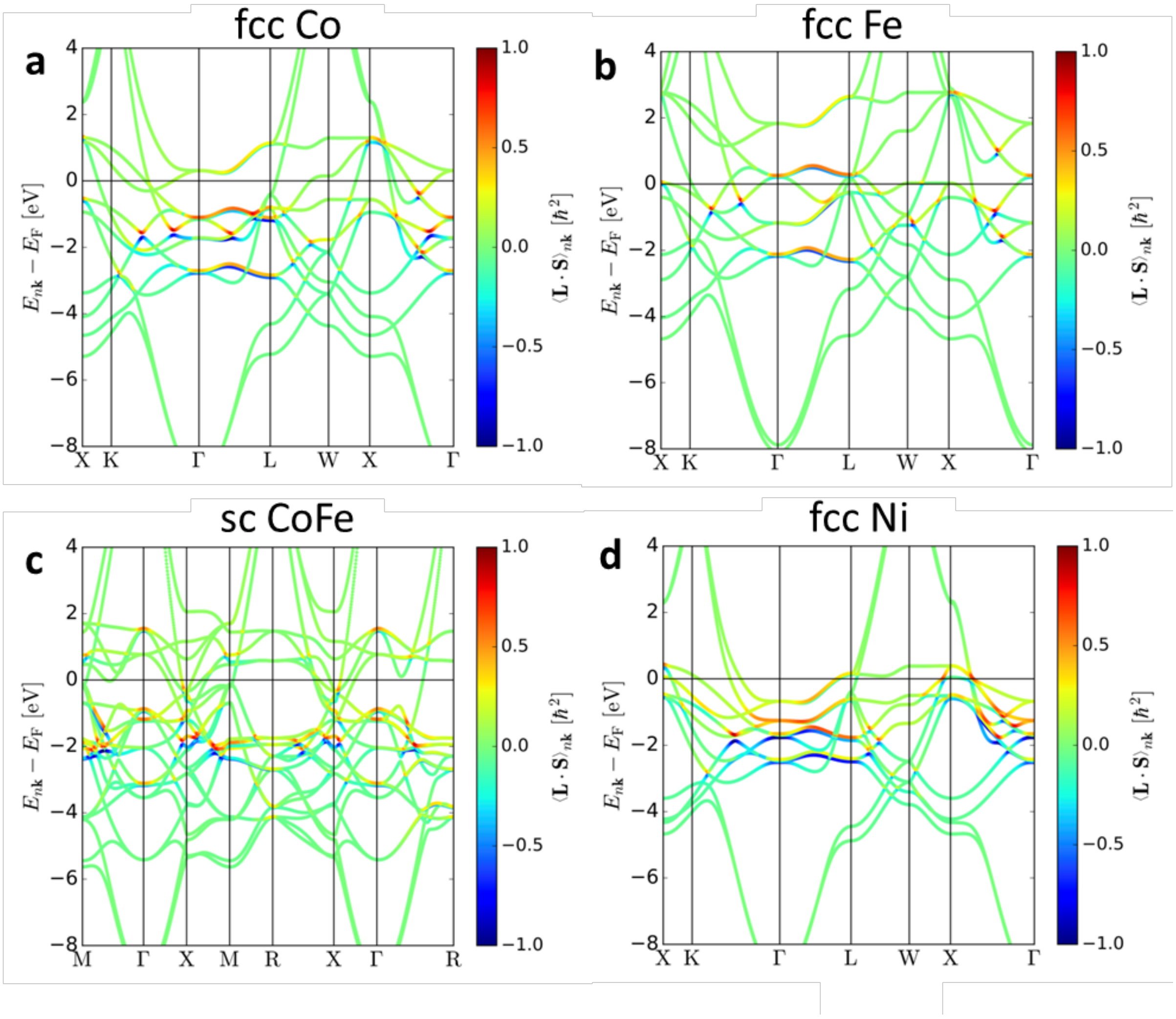

Fig. S2. Spin-orbit correlation $R_{FM} = \langle L \cdot S \rangle_{FM}$ for various FMs (Co, Fe, CoFe, and Ni). Spin-orbit correlation $\langle L \cdot S \rangle_{n\mathbf{k}}$ for an energy eigenstate with the crystal momentum **k** in the band *n* for fcc Co (**a**), fcc Fe (**b**), sc CoFe (**c**), fcc Ni (**d**). Note that in Ni, a hot spot for the positive correlation appears near E_F (on the X-Γ line). In contrast, no distinct correlation is observed near E_F for other FMs.

Section S2. FM-thickness dependence of ST-FMR signals

This section shows raw ST-FMR data for various FM thicknesses. Figures S3, S4, S6, S7, and S8 show the FM-thickness-dependent variation of the raw data for Ni(t_{Ni})/Pt, Ni(t_{Ni})/Ta, Ni(t_{Ni})/Cu/Ta, HfO_x/Ni(t_{Ni})/Pt, and HfO_x/Ni(t_{Ni})/Ta, respectively. The FM thickness dependence analyses for various FM/NM bilayers in Figs. 3 and 4 of the main text are based on these raw data. On the other hand, Figure S5 shows the insertion-layer-thickness-dependent variation of the raw data for Ni(7)/Cu(t_{Cu})/Ta. Figure 4B in the main text is based on these data.

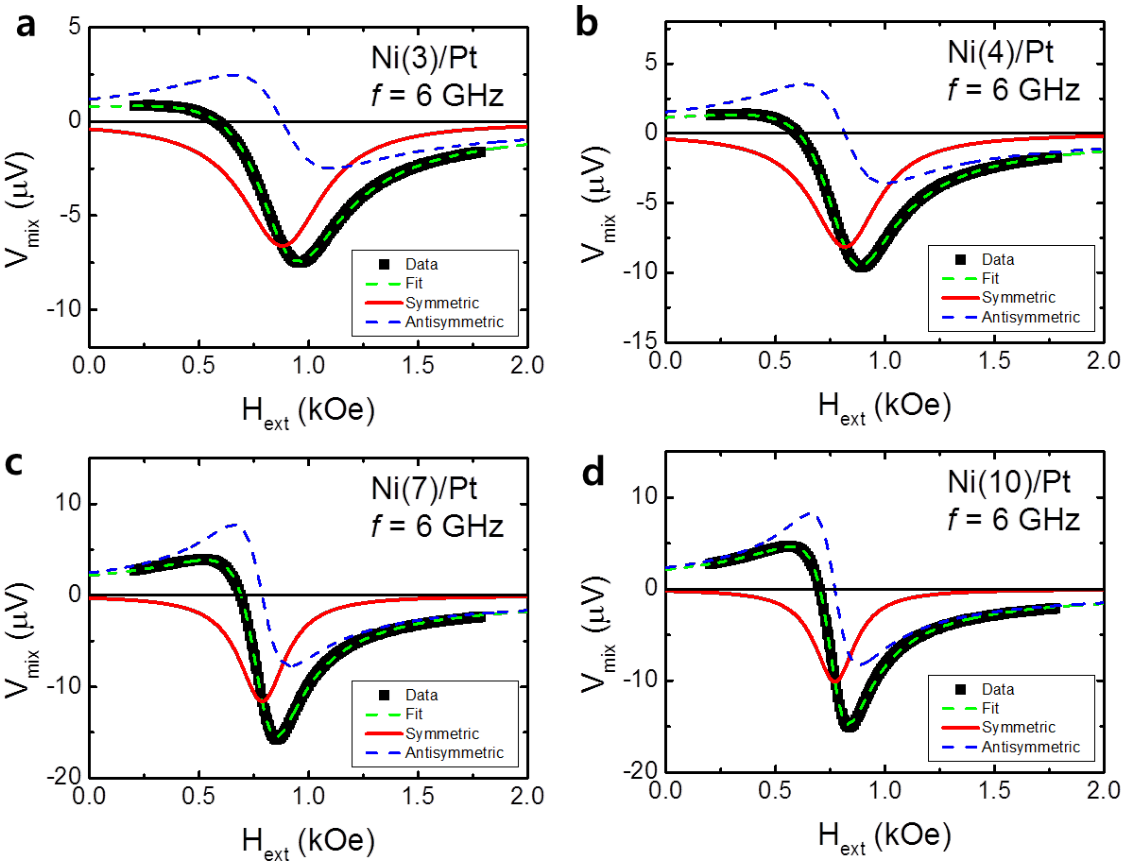

Fig. S3. $\boldsymbol{V_{mix}(H)}$ **of Ni($\boldsymbol{t_{Ni}}$)/Pt bilayers.** (**a-d**), t_{Ni} of (**a**) 3 nm, (**b**) 4 nm, (**c**) 7 nm, and (**d**) 10 nm. Symbols are experimental results, and lines are fitting results with Eq. (1).

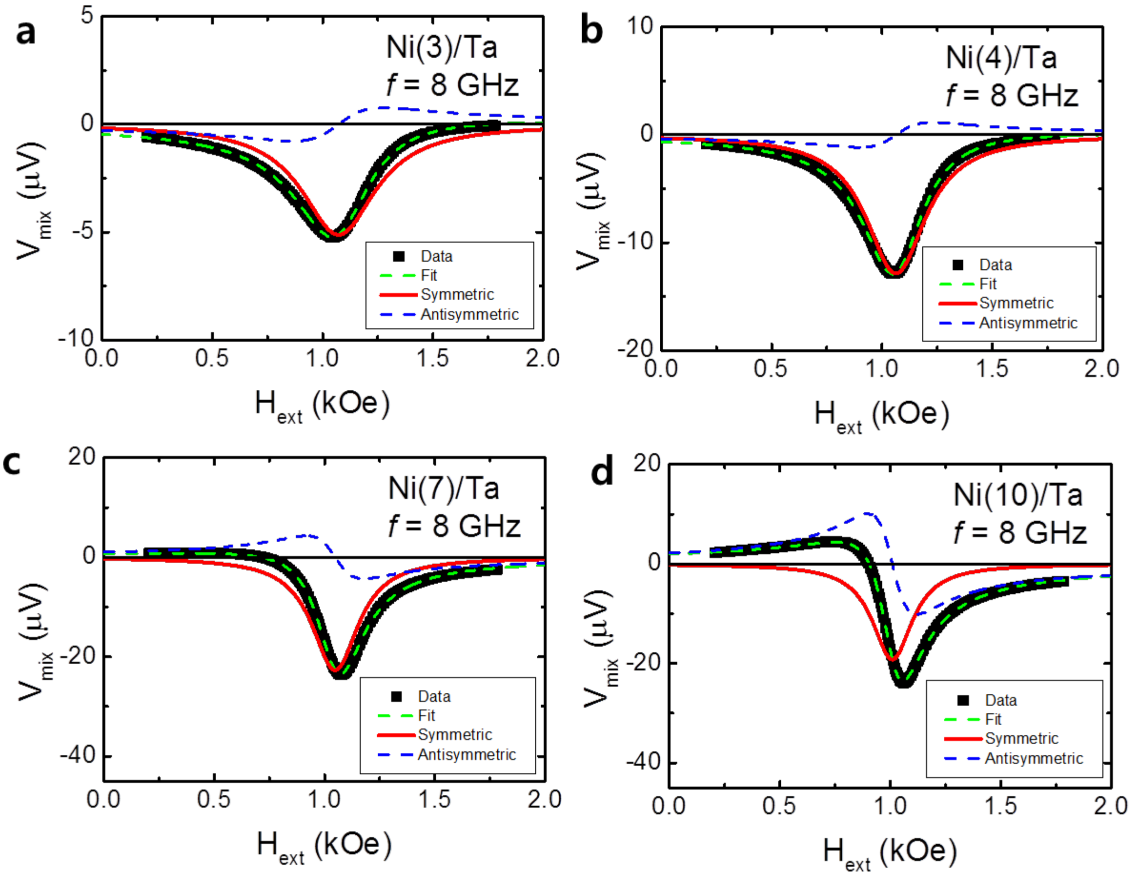

Fig. S4. $\boldsymbol{V_{mix}(H)}$ **of Ni(**$\boldsymbol{t_{Ni}}$**)/Ta bilayers. (a-d),** t_{Ni} of (**a**) 3 nm, (**b**) 4 nm, (**c**) 7 nm, and (**d**) 10 nm. Symbols are experimental results, and lines are fitting results with Eq. (1).

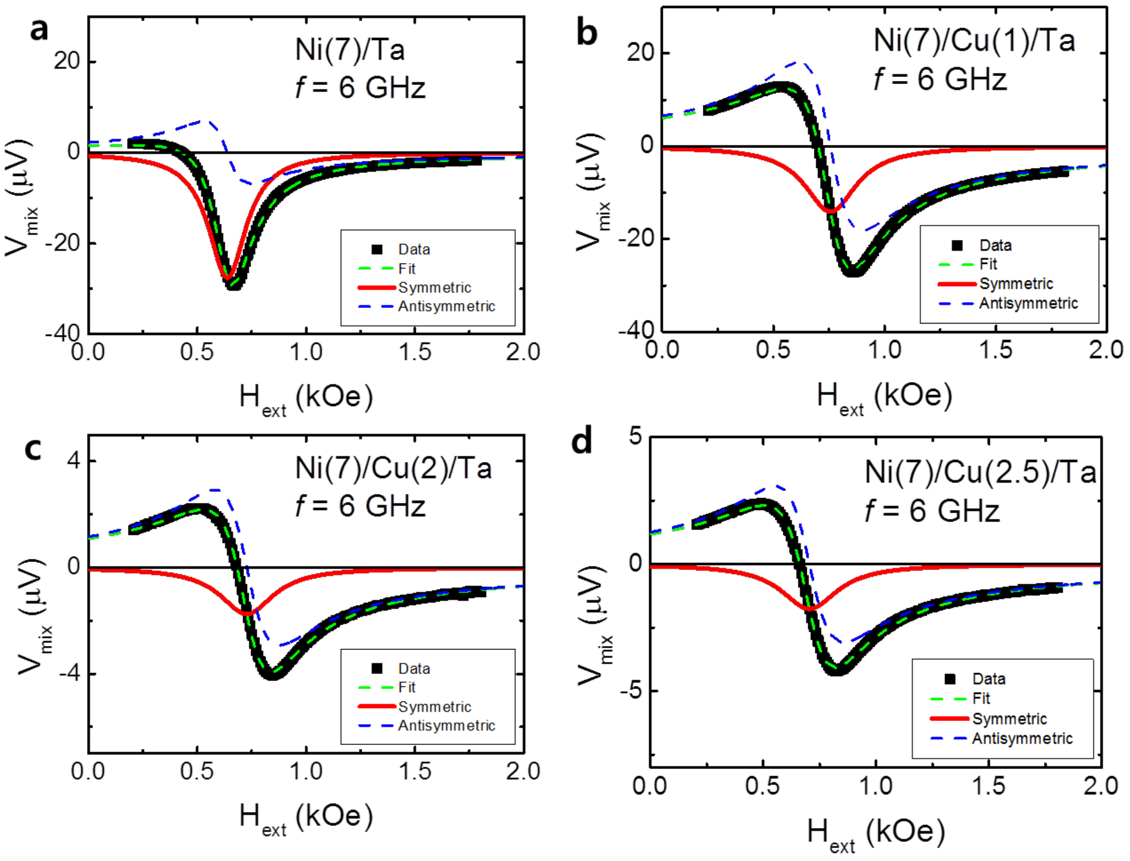

Fig. S5. $V_{mix}(H)$ **of Ni(7)/Cu(**t_{Cu}**)/Ta trilayers.** (**a-d**), t_{Cu} of (**a**) 0 nm, (**b**) 1 nm, (**c**) 2 nm, and (**d**) 2.5 nm. Symbols are experimental results, and lines are fitting results with Eq. (1).

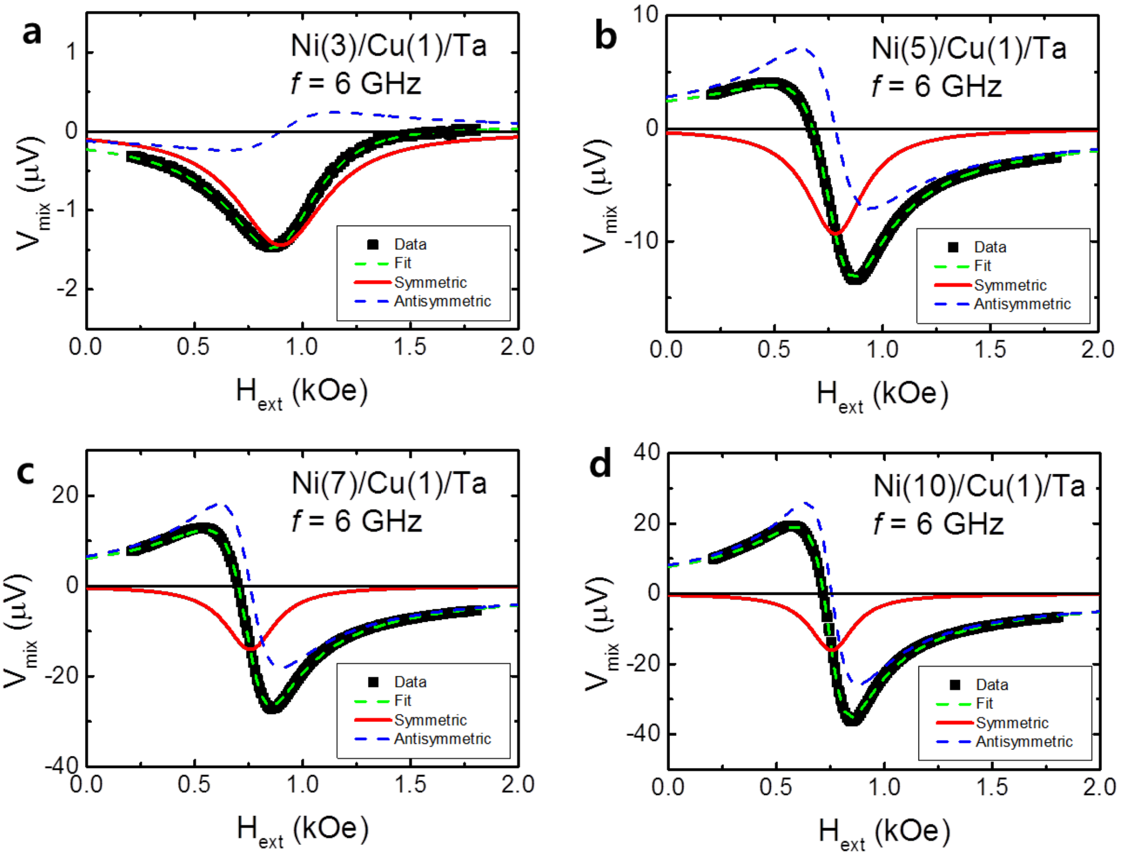

Fig. S6. $\boldsymbol{V_{mix}(H)}$ **of Ni($\boldsymbol{t_{Ni}}$)/Cu(1)/Ta trilayers. (a-d)**, t_{Ni} of (**a**) 3 nm, (**b**) 5 nm, (**c**) 7 nm, and (**d**) 10 nm. Symbols are experimental results, and lines are fitting results with Eq. (1).

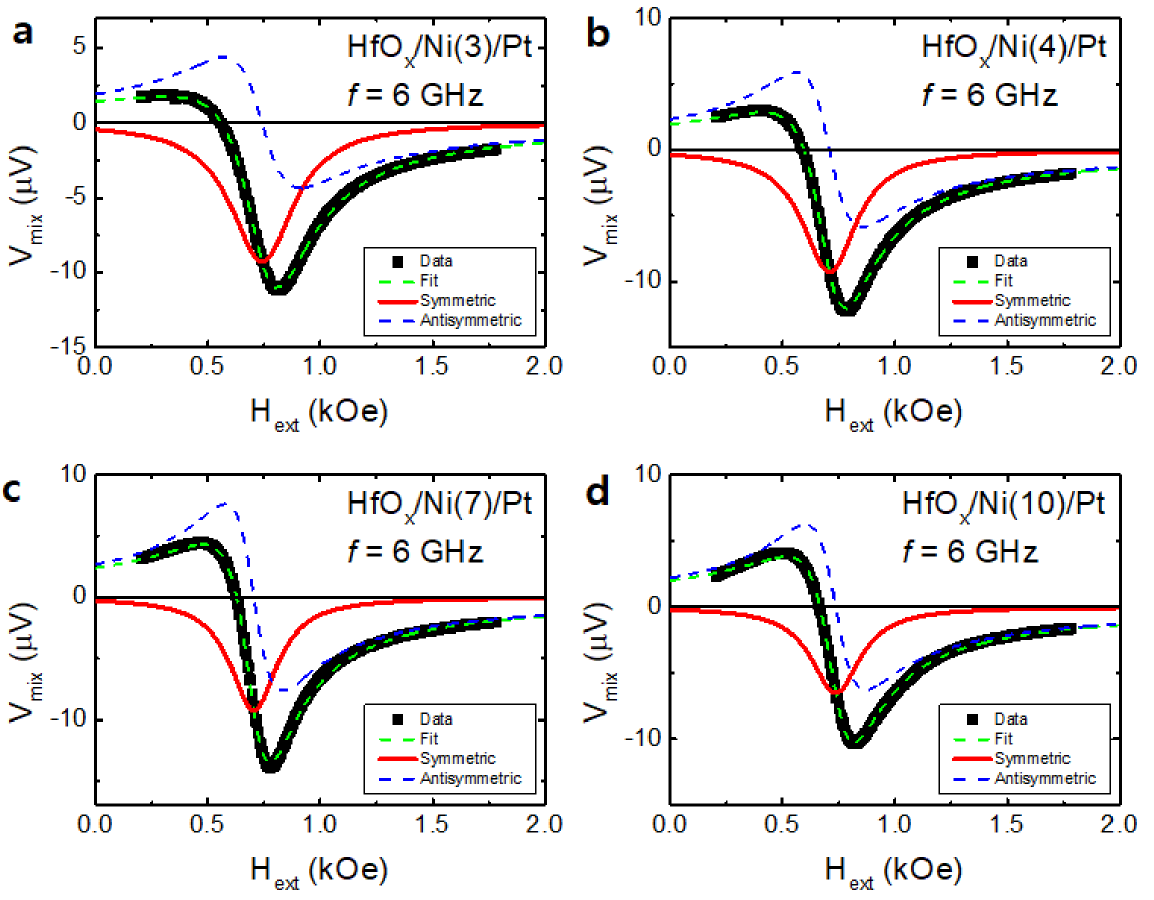

Fig. S7. $\boldsymbol{V_{mix}(H)}$ **of HfO$_x$/Ni($\boldsymbol{t_{Ni}}$)/Pt trilayers.** (**a-d**), t_{Ni} of (**a**) 3 nm, (**b**) 5 nm, (**c**) 7 nm, and (**d**) 10 nm. Symbols are experimental results, and lines are fitting results with Eq. (1).

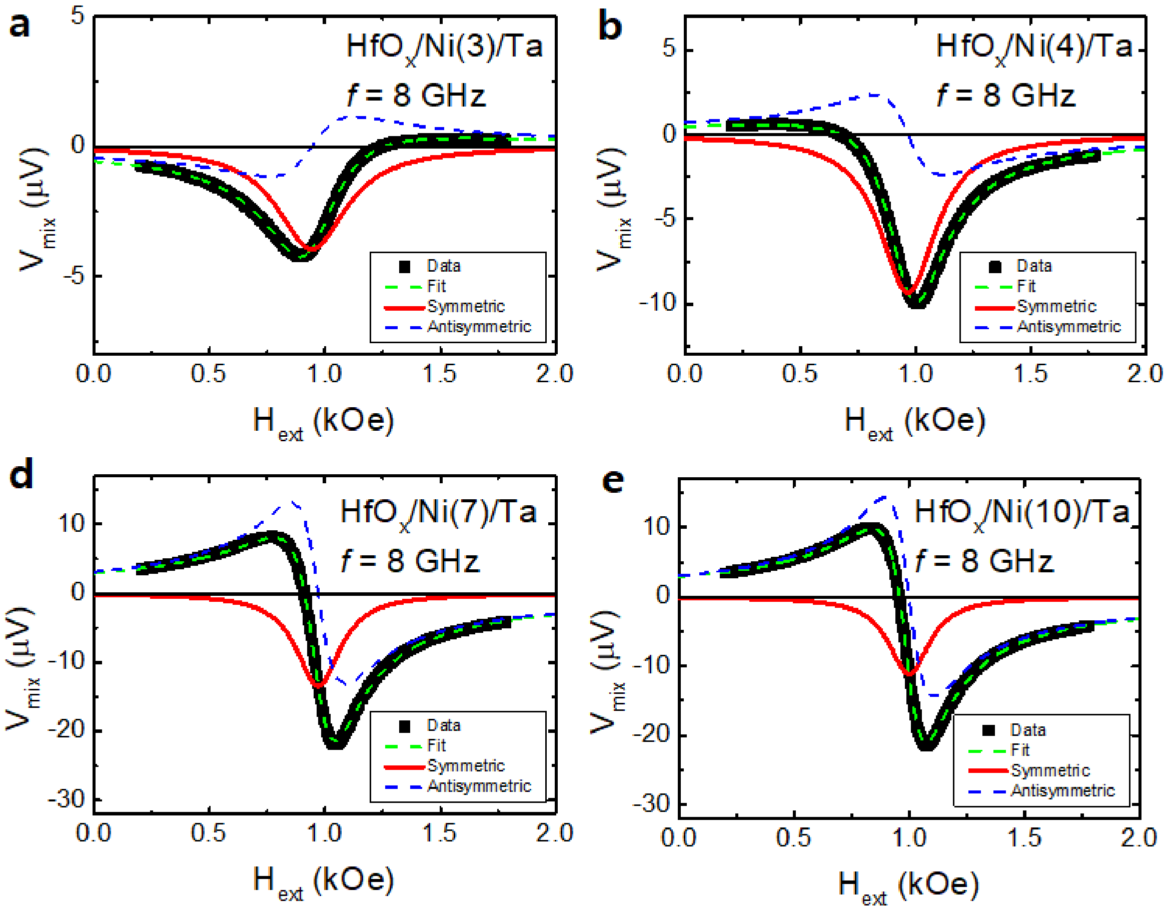

Fig. S8. $\boldsymbol{V_{mix}(H)}$ **of HfO$_x$/Ni($\boldsymbol{t_{Ni}}$)/Ta trilayers.** (**a-d**), t_{Ni} of (**a**) 3 nm, (**b**) 5 nm, (**c**) 7 nm, and (**d**) 10 nm. Symbols are experimental results, and lines are fitting results with Eq. (1).

Section S3. Linewidth (*ΔH*) modulation by a DC current

In addition to the ST-FMR, a few different measurement schemes are used to determine the sign of the effective spin Hall angle (θ_{DL}^{eff}) for Ni/Pt and Ni/Ta bilayers. This section describes one of such schemes; the measurement of the change of ST-FMR linewidth (*ΔH*) as a function of DC current (I_{dc}). According to the ST-theory, the spin current injected into the magnetic moment increases or decreases the effective magnetic damping parameter (α_{eff}) and hence *ΔH*, depending on its relative orientation to the magnetic moment (*23*). Therefore, it is possible to determine the sign of θ_{DL}^{eff} in the FM/NM bilayers by measuring the change in *ΔH* with respect to I_{dc}. In order to determine the sign of θ_{DL}^{eff}, we carry out ST-FMR measurements with varying I_{dc}.

Figure S9 presents the extracted *ΔH* as a function of I_{dc} for Ni(5)/Pt and Ni(5)/Ta samples at f = 7 GHz. In both samples, *ΔH* increases with increasing I_{dc} for $H > 0$ (φ = -135°) while the trend is opposite for $H < 0$ (φ = 45°). The results are in accord with Fig. 3 in that the sign of θ_{SH}^{eff} for Ni/Ta is the same as that for Ni/Pt. It confirms the abnormal sign of θ_{DL}^{eff} (> 0) for the Ni/Ta bilayer. Note that we obtain the same result for Ni(7)/Pt and Ni(7)/Ta samples (not shown).

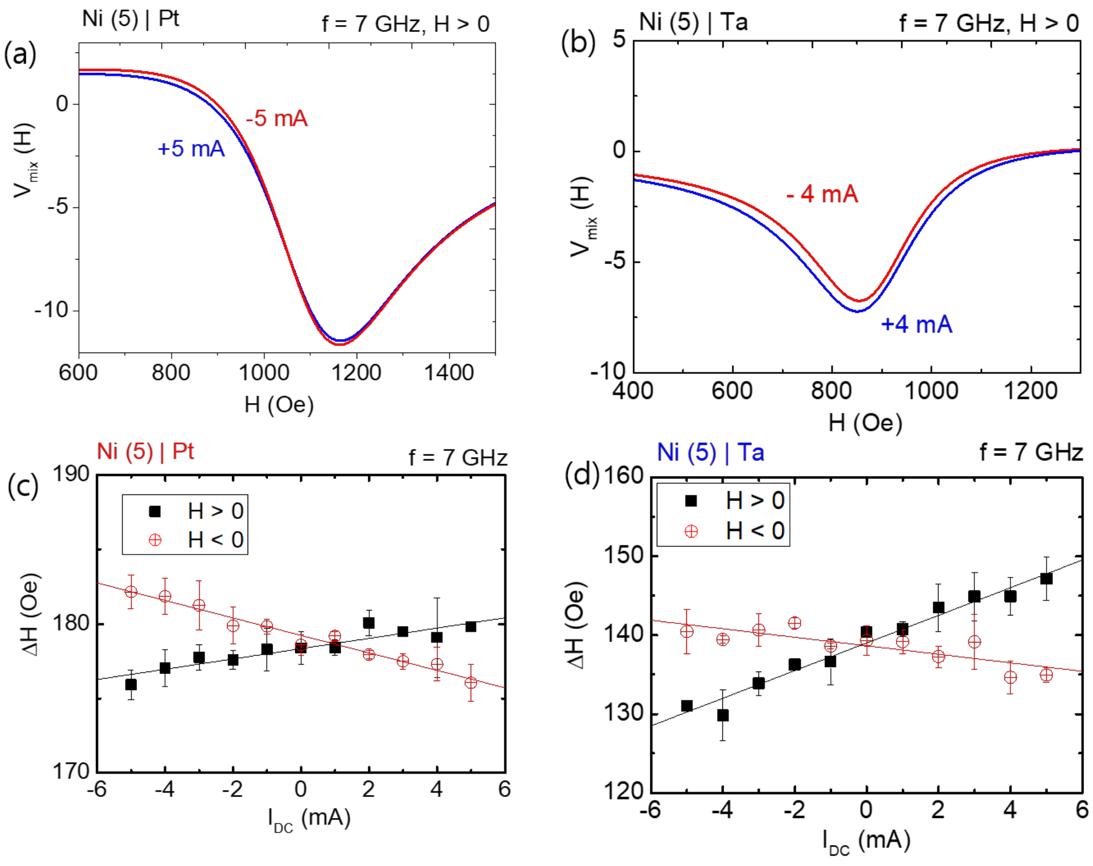

Fig. S9. Linewidth (*ΔH*) modulation by a DC current. Raw $V_{mix}(H)$ data of (**a**) Ni(5)/Pt and (**b**) Ni(5)/Ta bilayers. *ΔH* as a function of I_{DC} for (**c**) Ni(5)/Pt and (**d**) Ni(5)/Ta bilayers.

Section S4. In-plane 2nd harmonic Hall measurements

This section presents another experimental evidence of the abnormal (i.e. positive) DLT sign for the Ni/Ta bilayer, consistent with the ST-FMR measurement results. To this end, the 2nd harmonic Hall measurement has been performed for in-plane magnetization systems, where this method has been widely used to quantify SOTs.

We prepared the multilayer films, consisting of AlO_x(2)/FM/NM/substrate, by DC/RF magnetron sputtering at room temperature. The combinations of the FM and NM were Ni(3)/Ta(7.5), Co(3)/Ta(7.5), Ni(3)/Pt(5)/Ta(2), and Co(3)/Pt(5)/Ta(2) (nominal thickness in nm). Photolithography and ion milling were used to pattern Hall bar devices. The width of the Hall bar is 10 μm. The AC current of 10 mA at the frequency of 11 Hz was applied along the *x*-direction during the simultaneous measurement of the 1st harmonic Hall resistance response (R_H^{ω}) and the 2nd harmonic response ($R_H^{2\omega}$). An external magnetic field (H_{ext}) was applied at an angle (φ) in the direction of current in the film plane. The measurements were repeated for various values of H_{ext} from 2.5 kOe to 20 kOe. In the presence of SOTs, the AC current induces quasi-static magnetic oscillations and thus periodic changes in the Hall resistance. In addition, since the AC current induces a periodic temperature gradient (∇T), and Joule heating is quadratic to the current, the 2nd harmonic response can also be affected by thermal effects. In the measurement in which a B_{ext} rotates in the film plane, the heating contribution has the same angular dependence at the $R_H^{2\omega}$ to the DLT. The R_H^{ω} and $R_H^{2\omega}$ can be written as[5]

$$R_H^{\omega}(\theta,\varphi) = R_{AHE}\ cos(\theta) + R_{PHE}\ sin^2(\theta)sin(2\varphi) \quad \text{(S7)}$$

$$R_H^{2\omega}\left(\theta = \frac{\pi}{2},\varphi\right) = A\ \ cos(\varphi) + B\ \left(2cos^3(\varphi) - cos(\varphi)\right) \quad \text{(S8)}$$

where

$$A = \left(\frac{R_{AHE}}{H_{ext}+H_{demag}-H_{ani}}\right)H_{DL} + I_o\alpha\nabla T\ \ , \quad B = \left(\frac{2R_{PHE}}{H_{ext}}\right)(H_{FL} + H_{Oe}).$$

Here, $R_{AHE(PHE)}$ is the anomalous (planar) Hall resistance, H_{demag} is the out-of-plane demagnetization field, and H_{ani} is the in-plane magnetic anisotropy field, H_{Oe} is the Oersted field, $H_{DL(FL)}$ is the effective field due to the DLT(FLT), I_o is the amplitude of AC current and α is the Nernst coefficient.

Figure S10 shows the $R_H^{2\omega}(\varphi)$ obtained from the four devices in which all of $R_H^{2\omega}$ vs φ can be well fitted by Eq. (S8). We also obtained the values of R_{AHE} and $H_{demag} - H_{ani}$ for all samples from the anomalous Hall responses (see Fig. S11), where the R_H^{ω} was measured as a function of a magnetic field applied perpendicular to the plane (H_z). Note that the sign of R_{AHE} for the Ni | Ta is the same as that for the Ni | Pt, but opposing to those for the Co | Ta and the Co | Pt.

As a result, A and A / $R_{\rm AHE}$ are summarized in Fig. S12 as a function of H_{eff}^{-1} where $H_{eff} = H_{ext} + H_{demag} - H_{ani}$. According to Eq. (S8), the sign of H_{DL} can be determined by the sign of the slope in the A / $R_{\rm AHE}$ vs H_{eff}^{-1}. As shown in the figure, one can conclude that the DLT sign for Ni | Ta is the same as those for Ni/Pt and Co/Pt, but opposite to one for Co/Ta. Our 2nd harmonic measurements are consistent with the ST-FMR measurements in that the DLT sign for Ni/Ta is abnormal (positive), but the DLT signs for Ni/Pt, Co/Ta, and Co/Pt are normal.

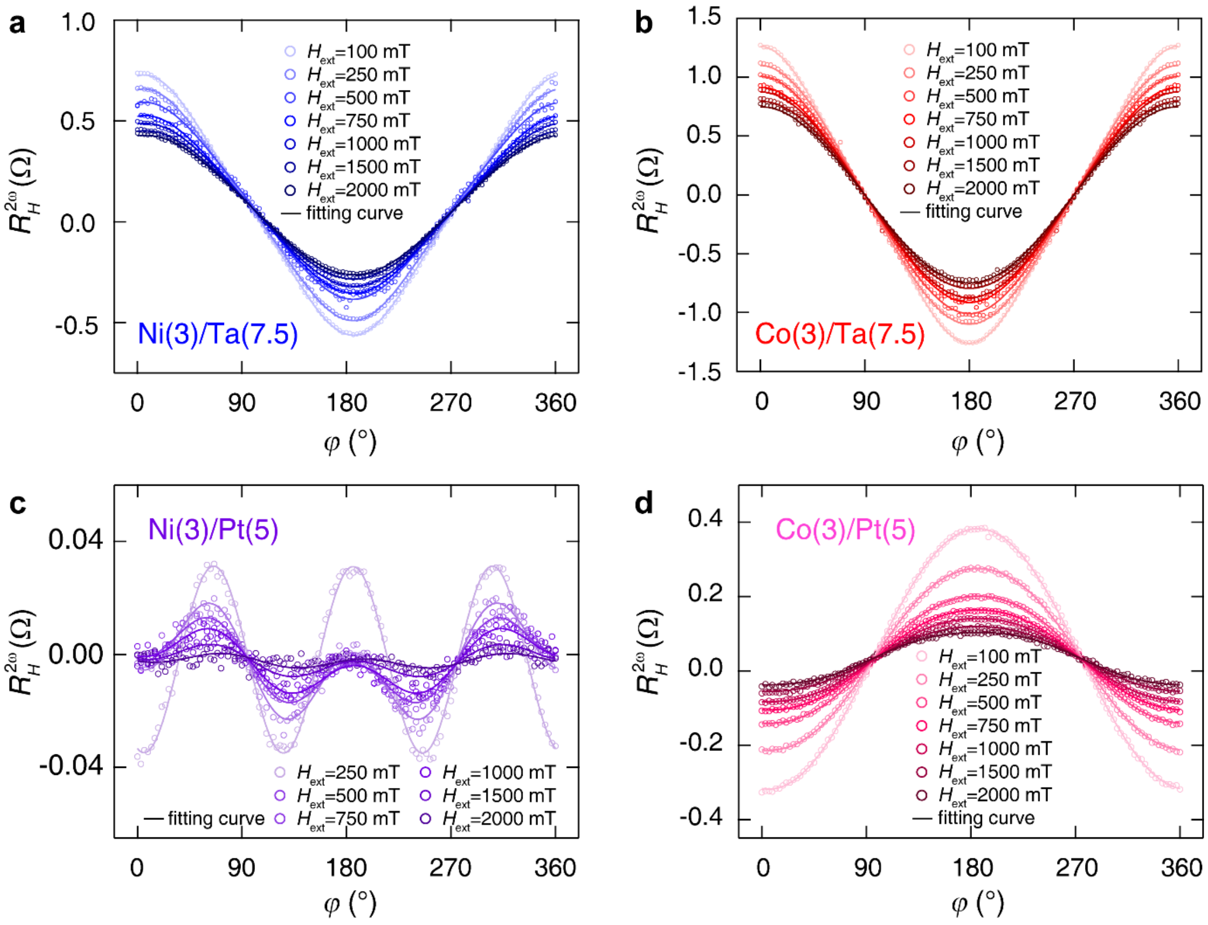

Fig. S10. $R_H^{2\omega}(\varphi)$ **of FM/NM layers.** (**a**) Ni(3)/Ta(7.5), (**b**) Co(3)/Ta(7.5), (**c**) Ni(3)/Pt(5), and (**d**) Co(3)/Pt(5).

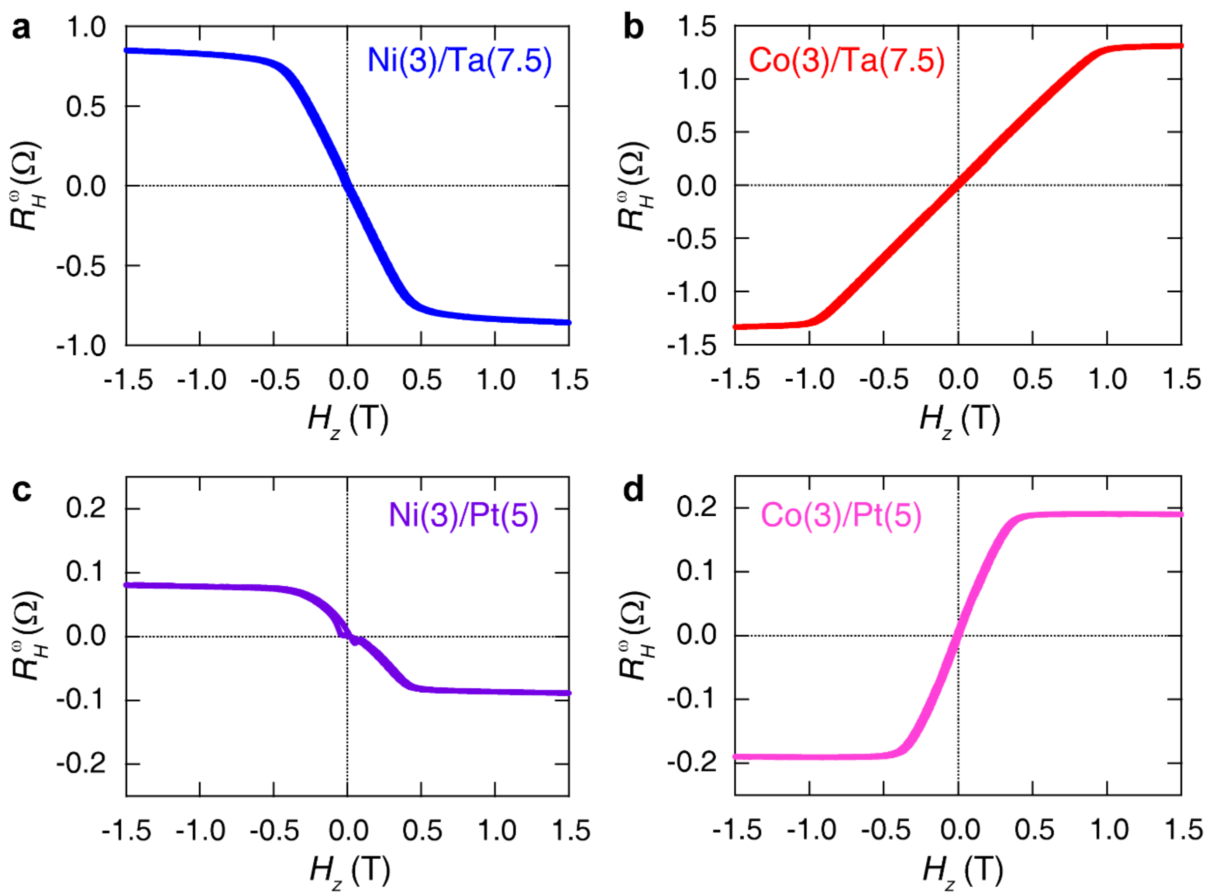

Fig. S11. $\boldsymbol{R_H^{\omega}}$**(H$_z$) of FM/NM layers.** (**a**) Ni(3)/Ta(7.5), (**b**) Co(3)/Ta(7.5), (**c**) Ni(3)/Pt(5), and (**d**) Co(3)/Pt(5).

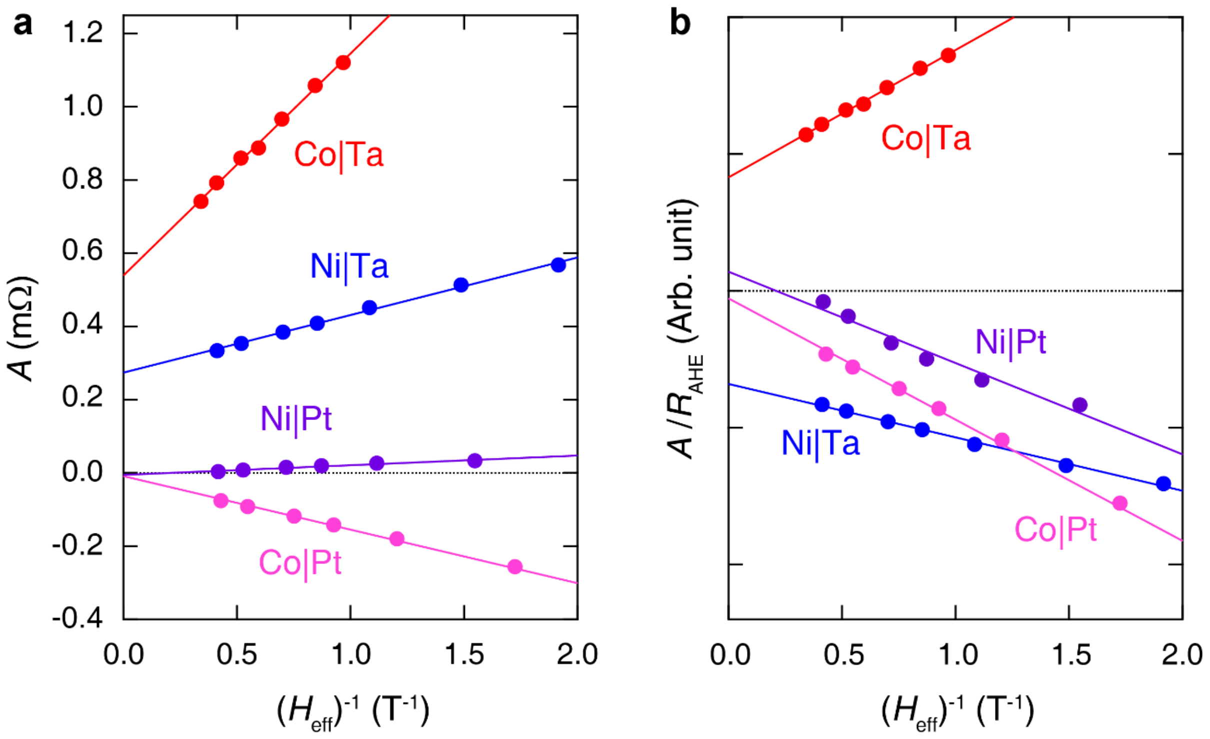

Fig. S12. Estimation of SOTs from 2nd harmonic Hall measurements. Obtained (**a**) A and (**b**) A / R_{AHE} as a function of H_{eff}^{-1}, where $H_{eff} = H_{ext} + H_{demag} - H_{ani}$.

Section S5. Discussion on the SOC in 3d-FM

We evaluated the Landé *g*-factors for Ni/Pt, Ni/Ta, CoFeB/Pt and CoFeB/Ta from the ST-FMR spectra using the Kittel formula. It is well known that the *g*-factor should be 2.0 if the orbital magnetic moment is completely quenched by the crystal field. Thus the deviation of the *g*-factor from 2.0 implies the unquenched orbital magnetic moment[6]. Moreover, the deviation of the *g*-factor from 2.0 can be regarded as a measure of how "friendly" a given FM is to an injected orbital current. According to Fig. S13, the deviation (*g*-factor – 2.0) is on average 0.22 for Ni/Pt, 0.17 for Ni/Ta, 0.09 for CoFeB/Pt, and 0.09 for CoFeB/Ta. Thus the deviation is about two times larger when FM is Ni than when FM is CoFeB. This result is consistent with our result for the DLT measurement, which indicates that the OHE is more important when FM is Ni than when FM is CoFeB. The larger deviation for Ni than CoFeB may be partially attributed to the fact that Ni has a larger spin-orbit coupling (SOC) than CoFeB, and larger SOC makes the orbital-to-spin conversion more efficient.

Regarding the origin of the abnormal sign in Ni/Ta, we point out two special features of this bilayer system. First, the spin Hall conductivity and the orbital Hall conductivity have the opposite signs in Ta [Ref. 10 for bcc α-Ta and our own calculation (section S1) for A15-structured β-Ta]. Thus Ta is the material where the SHE and the OHE tend to generate the torque of the opposite signs. The Hund's rule provides an intuitive explanation for this sign difference, which is verified by the extensive tight-binding calculation (Ref. 10) and our own first-principles calculation (section S1). The second special feature of Ni/Ta arises from Ni. In order for a large torque to arise from the injection of the orbital current to an FM, one desires an FM that does not suppress the orbital magnetic moment strongly and does allow efficient conversion of the orbital angular momentum to the spin angular momentum through the SOC. Among the 3*d* FMs, Ni has the largest orbital magnetic moment and the largest SOC[7,8]. These

features make Ni/Ta an excellent system to realize the torque sign reversal by the OHE. We note that the above qualitative explanation is indeed in agreement with our theoretical and experimental results.

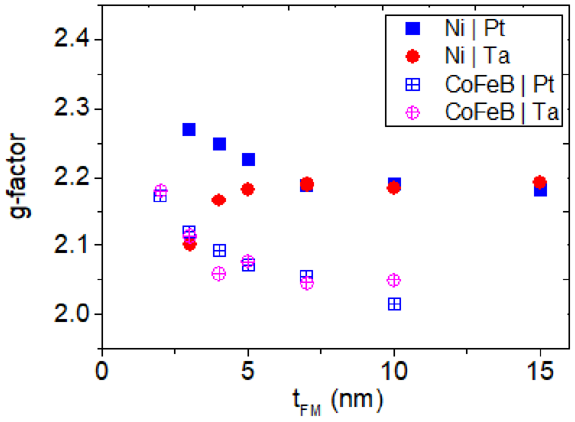

Fig. S13. Evaluated Landé g-factors. Results for Ni/Pt, Ni/Ta, CoFeB/Pt, and CoFeB/Ta are presented, which are evaluated from the ST-FMR spectra using the Kittel formula.

Section S6. Overall trends of the SOT variation

According to our theoretical calculation (Fig. 2C), the effective spin Hall conductivity for Pt-based bilayers is positive and varies little as FM changes from Fe to CoFe, Co, and Ni, whereas the effective spin Hall conductivity for Ta-based bilayers changes from negative to positive as FM changes from Fe to CoFe, Co, Ni. Our ST-FMR measurement combined with the FM thickness-dependence analysis [Eq. (3)] confirms this trend for FeB/Pt, CoFeB/Pt, Co/Pt, Ni/Pt, and for FeB/Ta, CoFeB/Ta, Co/Ta, Ni/Ta, as summarized in Fig. S14 (also Figs. 3H and 3I). The composition of CoFeB is $Co_{40}Fe_{40}B_{20}$ and the composition of FeB is $Fe_{80}B_{20}$. The overall trend of the measured effective spin Hall conductivity variation among these devices is in agreement with the theoretical prediction.

One comment is in order. We note that there is a big uncertainty in the SHA of the Co/Ta bilayer [Fig. S14(a)] since it was evaluated by the method suggested in Sec. S5 due to the following reason. For the Co/Ta bilayer, the SHA evaluation via the Eq. (3) was difficult since Eq. (3) assumes magnetic properties of a FM material in a given FM/NM bilayer to be independent of the FM thickness. In contrast, we observed a large in-plane magnetic anisotropy and a giant enhancement of the magnetic damping in the thin Co thickness range (< 5 nm) of the Co/Ta bilayer (not shown). We surmise that magnetic property change with the Co thickness may be due to the giant strain effect arising from the lattice mismatch at the Co/Ta interface. For instance, a previous report[9] discussed such issues and possible influences on the spin dynamics. However, we do not clearly understand the reason for the thickness dependence.

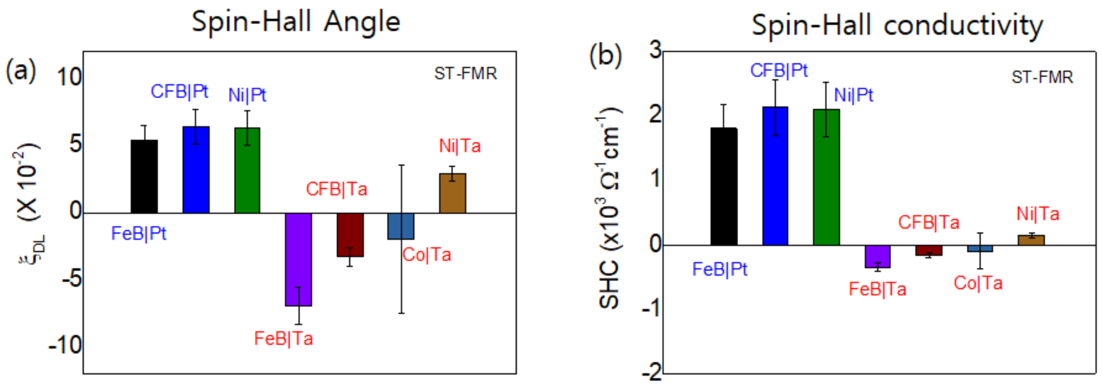

Fig. S14. The trend of SOT variation among various FM/NM bilayers. (**a**) The damping-like torque efficiency ξ_{DL} (or effective spin Hall angle) and (**b**) the effective spin Hall conductivity for various FM/NM bilayers with FM=FeB, CoFeB, Co, Ni, and NM=Pt, Ta. The error bars in (a) and (b) indicate single-standard-deviation uncertainties from the fitting.

---

[1] Blügel, S. & Bihlmayer, G. Full-potential linearized augmented planewave method. In J. Grotendorst, S. Blügel, D. Marx (eds), Computational Nanoscience: Do It Yourself! vol. 31, 85, http://www.flapw.de (John von Neumann Institute for Computing, Jülich, 2006).

[2] Wimmer, E., Krakauer, H., Weinert, M. & Freeman, A. J. Full-potential self-consistent linearized-augmented-plane-wave method for calculating the electronic structure of molecules and surfaces: O2 molecule, Phys. Rev. B 24, 864 (1981).

[3] Perdew, J. P., Burke, K. & Ernzerhof, M. Generalized Gradient Approximation Made Simple. Phys. Rev. Lett. 77, 3865 (1996).

[4] Pizzi G. et al., Wannier90 as a community code: new features and applications, J. Phys. Cond. Matt. 32, 165902 (2020).

[5] Avci, C. O. et al. Interplay of spin-orbit torque and thermoelectric effects in ferromagnet/normal-metal bilayers. Phys. Rev. B 90, 224427 (2014).

[6] Chikazumi, S. Physics of Ferromagnetism 2nd edition (Oxford University Press, 1997).

[7] Stohr, J. & Siegmann, H. C. Magnetism: From Fundamentals to Nanoscale Dynamics. Springer (2006).

[8] Papaconstantopoulos, D. A. Handbook of the Band Structure of Elemental Solids, Second Edition, Springer, New York (2015) and Inorg. Chem. vol 9, no8, 1898–1902 (1970).

[9] Takaçc, M. et al., Interfacial structure dependent spin mixing conductance in cobalt thin Films, Phys. Rev. Lett. 115, 056601 (2015).

[10] Tanaka T., Kontani H., Naito M., Naito T., Hirashima D. S., Yamada K., Inoue J. Intrinsic spin Hall effect and orbital Hall effect in4dand5dtransition metals. Phys. Rev. B **77**, 165117 (2008).
